# Prognostic Role of Diastolic Left Ventricular Mechanical Dyssynchrony by Gated Single Photon Emission Computed Tomography Myocardial Perfusion Imaging in Post-Myocardial Infarction

**DOI:** 10.1055/s-0043-1764304

**Published:** 2023-05-01

**Authors:** Le Ngoc Ha, Nguyen Thi Thanh Trung, Mai Hong Son, Do Van Chien, Jin Chun Paeng

**Affiliations:** 1 Department of Nuclear Medicine, Hospital 108, Hanoi, Vietnam; 2 Department of Cardiology, Thai Binh Medical University, Thai Binh, Vietnam; 3 Department of Cardiology, Heart Institute, Hospital 108, Hanoi, Vietnam; 4 Department of Nuclear Medicine, Seoul National University Hospital, Seoul, Korea

**Keywords:** diastolic, left ventricular mechanical dyssynchrony, GATED SPECT, MPI, post-MI

## Abstract

**Objective** This study is aimed to assess the prognostic value of diastolic left ventricular mechanical dyssynchrony (LVMD) measured by gated-single photon emission computed tomography (GSPECT) myocardial perfusion imaging (MPI) in post-myocardial infarction (MI).

**Subjects and Methods** The study was conducted on 106 post-MI from January 2015 to January 2019. First, the indices of diastolic LVMD phase standard deviation (PSD) and histogram bandwidth (HBW) of post-MI were measured using the Cardiac Emory Toolbox. Subsequently, the post-MI patients were followed up, and the primary endpoint was major adverse cardiac events (MACEs). Finally, the prognostic value of dyssynchrony parameters for MACE was analyzed by the receiver-operating characteristics curve and survival analyses.

**Results** With the cut-off values of 55.5 degrees of PSD, the sensitivity and specificity in prediction of MACE were 75% and 80.8%, with the cut-off values of 174.5 degrees of HBW, the sensitivity and specificity were 75% and 83.3% respectively. There was a significant difference of time to MACE between groups of PSD less than 55.5 degrees and more than 55.5 degrees. PSD, HBW, and left ventricle ejection fraction (LVEF) assessed on GSPECT were significant factors in the prediction of MACE.

**Conclusion** Diastolic LVMD parameters of PSD and HBW derived from GSPECT are significant prognostic factors in predicting MACE in post-MI patients.

## Introduction

Left ventricular mechanical dyssynchrony (LVMD) is defined as the disparity in regional contraction timing, which can be a cause of decreased ejection function. LVMD is a consequence of various cardiac diseases including coronary artery disease (CAD) and myocardial infarction (MI). The role of LVMD has been investigated for several decades and significant prognostic implications of LVMD were reported across a variety of diseases. 1 2 Mollema et al showed that LVMD immediately after acute MI is a strong prediction marker of left ventricular remodeling at 6 months of follow-up. 3 After non-ST-elevation MI (STEMI), impaired left ventricular function independently predicted LVMD and the composite score had a prognostic value for identifying patients with persistently dilated and impaired LV function on follow-up. 4 LVMD is a stronger predicting factor than electrical dyssynchrony for deaths of all causes in patients with CAD. 5 In addition, diastolic LVMD presented incremental prognostic values in patients with left ventricular ejection fraction (LVEF) greater than 35%. 6

Currently, advances in image processing software allow to assess diastolic LVMD on gated single photon emission computed tomography (GSPECT) myocardial perfusion imaging (MPI), which are supposed to be more objective than those on tissue Doppler imaging. 7 Previous studies have focused on the association between diastolic LVMD and outcomes in patients who underwent cardiac resynchronization therapy (CRT). 8 9 A recent study reported that diastolic LVMD assessed by GSPECT MPI seems to have an association with cardiovascular mortality in CAD patients. 6 Although recent software can assess diastolic LVMD on GSPECT MPI easily but there are significantly fewer data concerning its prognosis in post-MI. Therefore, the aim of this study was to assess the diastolic LVMD by GSPECT MPI and its prognostic role in post-MI patients.

## Materials and Methods

### Study Population

The study was conducted on 106 post-MI patients who admitted to our institution from January 2015 to January 2019. MI was diagnosed according to the third universal definition of MI 10 and the European Society of Cardiology guidelines. 11 Inclusion criteria were (1) patients on recovery phase from acute MI at least 14 days after the symptom onset, (2) stable clinical and hemodynamic status, (3) normal range of all cardiac enzyme tests, and (4) ability to undergo rest GSPECT MPI. Patients with mechanical complications of MI (left ventricular free-wall rupture, ventricular septal rupture, papillary muscle rupture, pseudoaneurysm, and true aneurysm), life-threatening arrhythmias, atrial fibrillation, and history of other cardiovascular diseases were excluded. Diastolic LVMD parameters were calculated for all patients in post-MI cohort.

The present study protocol was reviewed and approved by the Institutional Review Board of Central Military Hospital 108 (approval no. 165/QD-V108) and informed consent was obtained from each patient before being included in the study.

### GSPECT MPI

GSPECT MPI was performed using a dual-head gamma camera (Infinia, GE Healthcare, Milwaukee, WI, USA). A 2-day protocol was used to acquire stress (exercise or pharmacological) and rest MPI as described on the European Association of Nuclear Medicine guidelines. 12 Briefly, GSPECT was recorded using a dual-head gamma camera equipped with low-energy high-resolution collimators 1 hour after intravenous injection of ^99m^ Tc-Sestamibi (0.31 mCi/kg). Electrocardiogram-gated images were obtained using eight frames/second. SPECT image was acquired using a step-and-shoot method for 30 seconds per step, with 6-degree interval. Images were reconstructed on 128 × 128 matrices. Raw data of rest MPI were processed using the Myovation Evolution software (Xeleris 3.0, GE Healthcare, Milwaukee, WI, USA). Quantitative analyses of perfusion and LVEF were performed using the QPS/QGS 2012 software (Cedars-Sinai Medical Center, Los Angeles, California). Summed rest score (SRS) greater than 13 was defined as severe perfusion defect, and LVEF of 40% or less was defined as severely decreased LVEF.

On GSPECT MPI, phase analysis was performed using the Emory Cardiac Toolbox (Emory University, Atlanta, GA) integrated on vendor-supplied analysis package (Xeleris 3.0, GE Healthcare, Milwaukee, WI, USA) to derive diastolic LVMD as mentioned in a previous study. 13 A phase distribution that represents data on the degree of diastolic dyssynchrony was created and displayed in polar map and histogram. The quantitative parameters for diastolic LVMD were derived from the phase histogram, and phase standard deviation (PSD, degrees) and histogram bandwidth (HBW, degrees) were calculated.

### Clinical Data and Follow-up

Post-MI patients were followed up within 12 months after discharging from hospital. Complete history taking, physical examination, laboratory test, and electrocardiogram were performed. The primary endpoint was major adverse cardiovascular event (MACE). Currently, MACE is defined as cardiac death, MI, and hospitalization due to heart failure. 14 Mortality and its cause were determined by telephone interviews with family members and/or medical records of the hospital.

### Statistical Analysis

Commercial software packages were used for statistical analysis (SPSS v. 20.0, IBM Corp.; GraphPad Prism v8.0, GraphPad Software Inc.). The relationship between PSD, HBW. and ejection fraction (EF), summed rest scored (SRS) in post-MI was evaluated using Student's *t* -test. The optimal cut-off value, sensitivity, and specificity of each diastolic LVMD parameters for cardiac events prediction were calculated using receiver-operating characteristic (ROC) curve analysis, and diagnostic power was assessed using the area under the curve (AUC). Logistic regression analysis was used to determine the significant parameters of diastolic LVMD to predict MACE. Estimating for time to MACE was analyzed by using Kaplan–Meier methods. The significance threshold was set at *p* -value less than 0.05.

## Results

### Patient Characteristics

The general characteristics of patient population are shown in Table 1 . Among 106 post-MI patients, 52.8% of patients had heart failure of the New York Heart Association (NYHA) class II and there are only 25.5% and 4.7% of classes III and IV, respectively. The number of patients who underwent revascularization using percutaneous coronary intervention was higher than the one received medical treatment and coronary artery bypass graft surgery. During follow-up, there were 20 (18.9%) patients presented with MACE (6 cardiac death, 7 hospitalization due heart failure, and MI). In the post-MI group, there were significant relationships of PSD and HBW with LVEF and perfusion. PSD and HBW were significantly higher in the LVEF less than 40% and SRS greater than 13 groups. ( *p* < 0.001, Table 2 .)

**Table 1:** Clinical characteristics of study population ( *n* = 106)

Characteristic	Post-MI ( *n* = 106)
Age (y, mean ± SD)	65.4 ± 10.31
Gender	
Male, *n* (%)	17 (16.0%)
Female, *n* (%)	89 (84.0%)
Diabetes	26 (24.5%)
Hypertension	71 (67.0%)
NYHA class	
I	18 (17.0%)
II	56 (52.8%)
III	27 (25.5%)
IV	5 (4.7%)
LVEF (mean ± SD)	46.8 ± 14.3
QRS duration > 120 milliseconds	6 (5.6%)
Left bundle block	6 (5.7%)
STEMI	9 (8.5%)
Number of diseased vessel	
1	57 (53.8%)
2	42 (39.6%)
3	6 (5.7%)
Treatment	
Medical treatment	44 (41.5%)
Percutaneous coronary intervention	61 (58.1%)
Coronary artery bypass graft surgery	1 (0.95%)
MACE	20 (18.9%)

**Table 2:** Relationship between PSD, HBW, EF, and SRS in post-MI

	PSD		HBW	
	Value	*p* -Value	Value	*p* -Value
EF (ejection fraction)				
≤ 40%	66.2 ± 16.2	< 0.001*	219.2 ± 61.1	< 0.001*
> 40%	41.8 ± 16.1		129.6 ± 59.2	
Summed rest score (SRS)				
< 13	33.1 ± 18.0	< 0.001*	128.7 ± 64.4	< 0.001*
≥ 13	60.4 ± 18.1		196.6 ± 63.7	

*Significant difference.

### Prediction of MACE

**Fig. 1:**
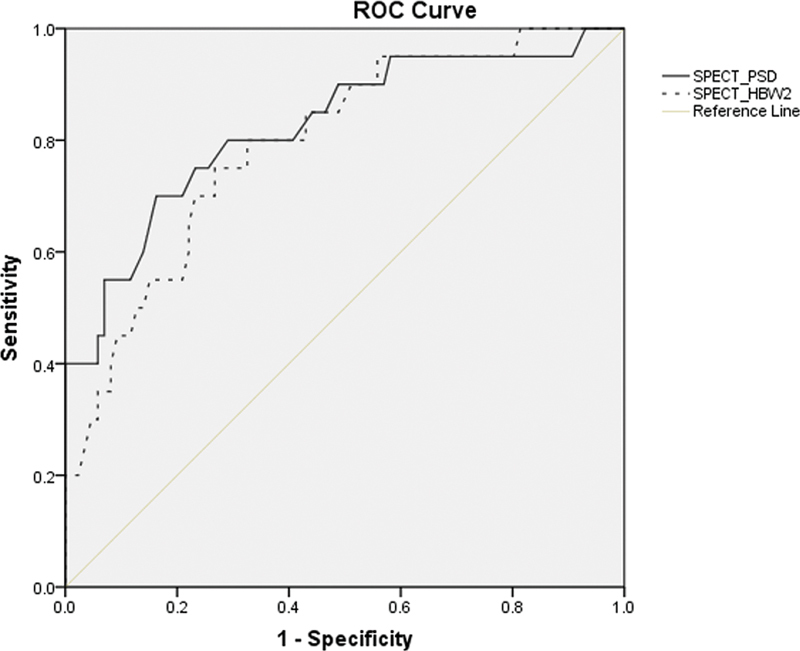
ROC curve of diastolic LVMD parameters to predict the cardiac death in a post-MI cohort.

**Fig. 2:**
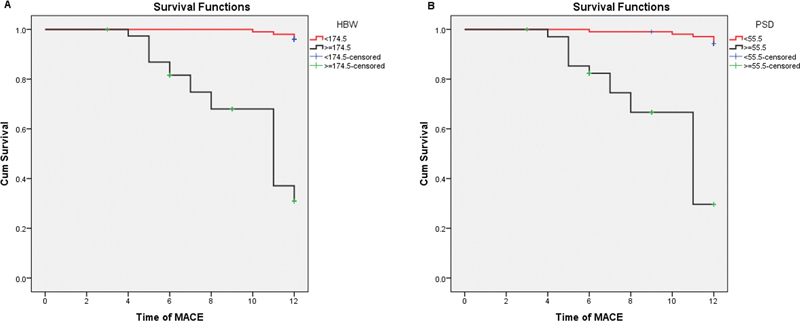
The Kaplan–Meier survival curve illustrated the time of MACE in patients with PSD < 55.5 and ≥ 55.5 degrees ( **A** ), HBW < 174.5 and ≥ 174.5 degrees ( **B** ).

**Fig. 3:**
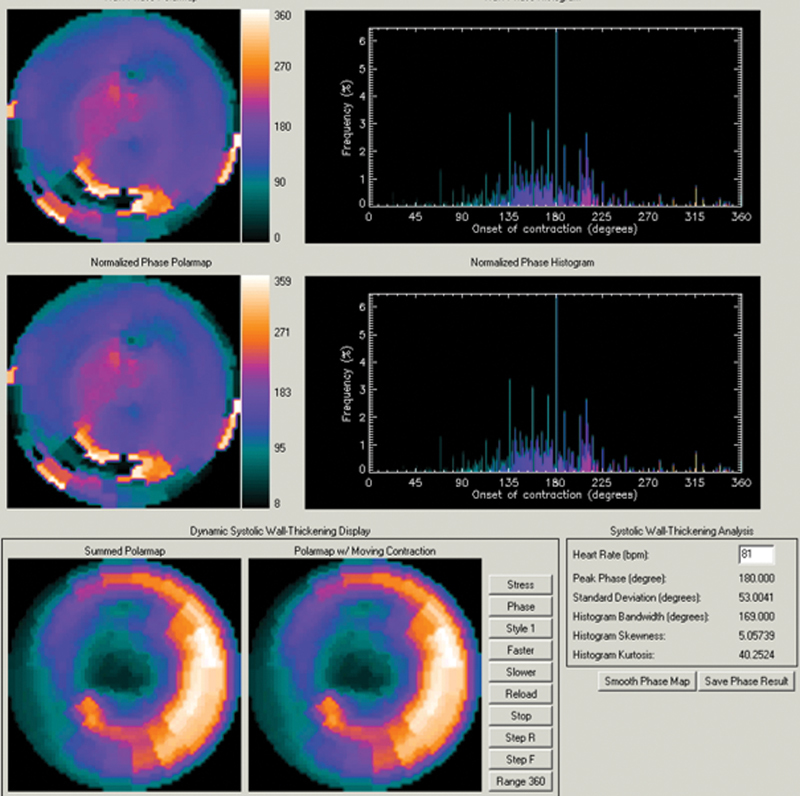
Assessment of LVMD by GSPECT MPI.

In the ROC curve analysis for MACE prediction, AUC of HBW was 0.851 (95% of CI, 0.772–0.931, *p* < 0.001) and AUC of PSD was 0.872 (95% of CI: 0.789–0.95, *p* < 0.001) ( Fig. 1 ). The optimal cut-off value of HBW for predicting MACE was 174.5 degrees. With this cut-off value, the sensitivity and specificity were 75.0% and 80.8%, respectively. The optimal cut-off value of PSD for predicting MACE was 55.5 degrees and the sensitivity and specificity were 75.0% and 83.3%, respectively. In univariate analysis, HBW, PSD, SRS, and LVEF were significant factors for predicting MACE and STEMI was not a significant factor for predicting MACE. In multivariate analysis, LVEF was the only significant factor for predicting MACE ( Table 3 ). The Kaplan–Meier curve showed the time to MACE stratified according to the optimal cutoff of PSD and HBW ( Fig. 2 ). The mean time of MACE in the group of PSD less than 55.5 degrees was 11.9 ± 0.07 months, which was significantly higher than 9.7 ± 0.15 of PSD of 55.5 degrees or greater ( *p* < 0.001, 95% of CI: 9.1–12.9). Similarly, the significant difference in the mean time of MACE was seen between HBW of 174.5 or greater and less than 174.5 degrees (12 vs. 9.8, *p* < 0.001, 95% of CI: 8.9–13.1). A representative case is shown in Fig. 3 .

**Table 3:** Logistic regression analysis for risk factors of MACE

	Univariate		Multivariate	
Factors	OR (95% CI)	*p* -Value	OR (95% CI)	*p* -Value
PSD	1.083 (1.045–1.122)	< 0.001*	1.054	0.090
HBW	1.017 (1.009–1.026)	< 0.001*	1.003	0.722
LVEF (> 40% vs. ≤ 40%)	11.252 (3.928–32.232)	< 0.001*	0.930	0.038*
SRS (> 13 vs. ≤ 13)	1.076 (1.018–1.137)	0.010*	0.968	0.461
STEMI	1.949 (0.230–16.538)	0.541	–	–

*Significant difference.

## Discussion

Our study reported the relationship between diastolic LVMD and EF, SRS derived from GSPECT MPI. It has been reported that the more diastolic LVMD is seen with the more perfusion defect due to MI, and the size of myocardial perfusion defect is one of independent predictors for LVMD. 15 16 Hamalainen et al reported that phase histogram parameters are correlated significantly with the extent of MI, infarct scar, and SRS; however, no statistically significant correlation was observed between summed stress score and diastolic LVMD parameters. 17

This study showed the value of PSD and HBW as the predictors for MACE of post-MI patients. Diastolic LVMD could predict the outcome in heart failure patients undergoing CRT and it is associated with adverse outcomes in CAD patients. 18 PSD and HBW of diastolic LVMD can predict the cardiac death with high sensitivity and specificity in a 12-month follow-up. On the ROC curve analysis, the areas under ROC curves for PSD and HBW to predict MACE were 0.83 and 0.77, respectively. Fudim et al showed that mortality in 5-year follow-up was estimated as 50.1% among CAD patients with LVEF less than 50% and diastolic LVMD. 6 The mortality of diastolic LVMD groups has been varying among studies, probably because it might depend on time of follow-up and the population characteristics.

Our study evaluated the predictive value of diastolic LVMD assessed by GSPECT MPI in post-MI patients. Tian et al reported that PSD and HBW were not predictors of cardiac mortality in patients who underwent to GSPECT MPI 3 months after MI. 19 However, Uebleis et al reported that diastolic LVMD, LVEF less than 20%, and summed rest score on GSPECT MPI were independent factors to predict cardiovascular mortality in advanced CAD patients. 20 Our study reported that diastolic LVMD indices (PSD and HBW) and LVEF were the independent factors to predict MACE in post-MI patients.

Recently, GSPECT MPI became a more popular approach than before, and it can be easily used to assess diastolic LVMD in patients with heart failure and CAD. A few previous studies have showed that high PSD and HBW indicating diastolic LVMD on GSPECT MPI can assist the clinicians to select appropriate candidates for CRT and assess the treatment response. 18 21 22 On phase analysis of GSPECT MPI, the cut-off values of 135 degrees for HBW and 43 degrees for PSD were effectively used to predict the response to CRT. 23 In a study including a large group of CAD patients, Hess et al reported that HBW was a stronger factor related to mortality than other parameters of diastolic LVMD. 5 However, in our study, both PSD and HBW were effective prognostic factors in predicting MACE. Our study is in line with a previous report that diastolic LVMD was a stronger association factor with causes of mortality in comparison with systolic LVMD. 24 Additionally, Zafrir et al reported that PSD can predict cardiac events and cardiac death in heart failure patients treated with implantable cardioverter defibrillator, 25 such as the efficacy of PSD in our study.

The limitation of this study is the time of follow-up was not long enough to estimate the overall survival. It was challenging to follow up the patients from different regions of country for long term because many patients could not be admitted to our hospital due to social distancing during the COVID-19 pandemic. In addition, the limited number of enrolling patients (106 post-MI patients) may impact the results of our study due to selection bias.

## Conclusion

Diastolic LVMD indices including PSD and HBW can be easily assessed by GSPECT MPI, and can be effective prognostic factors for predicting MACE.
